# COVID-19 vaccine acceptance and associated factors in 13 African countries: A systematic review and meta-analysis

**DOI:** 10.3389/fpubh.2022.1001423

**Published:** 2023-01-24

**Authors:** Jember Azanaw, Mastewal Endalew, Demisu Zenbaba, Eshetu Abera, Vijay Kumar Chattu

**Affiliations:** ^1^Department of Environmental and Occupational Health and Safety, Institute of Public Health, College of Medicine and Health Sciences, University of Gondar, Gondar, Ethiopia; ^2^Department of Public Health, School of Health Sciences, Goba Referral Hospital, Madda Walabu University, Bale Goba, Ethiopia; ^3^Department of Community Medicine, Faculty of Medicine, Datta Meghe Institute of Medical Sciences, Wardha, India; ^4^Department of Occupational Science and Occupational Therapy, Temerty Faculty of Medicine, University of Toronto, Toronto, ON, Canada; ^5^Center for Transdisciplinary Research, Saveetha Institute of Medical and Technological Sciences, Saveetha University, Chennai, India

**Keywords:** COVID-19 vaccine acceptance, associated factors, systematic review, meta-analysis, sub-Saharan Africa, COVID-19

## Abstract

**Background:**

The COVID-19 pandemic has severely affected the entire world, especially sub-Saharan Africa. As a result, researchers and government agencies are working to create effective COVID-19 vaccinations. While vaccination campaigns are moving rapidly in high-income nations, COVID-19 is still ruthlessly affecting people in low-income nations. However, this difference in the spread of the disease is not because of a lack of a COVID-19 vaccine but mainly due to people's reluctance. As a result, this review summarized the data on COVID-19 vaccination adoption and factors related among nations in sub-Saharan Africa.

**Method:**

Comprehensive searches were conducted using PubMed, Embase, Medline, Web of Science, Google Scholar, and the Cochrane Library databases. The risk of bias and methodological quality of each published article that fit the selection criteria were evaluated using Critical Appraisal Checklist tools. All statistical analysis was done by STATA 16.

**Results:**

This review was based on 29 studies with 26,255 participants from sub-Saharan Africa. Using a random-effects model, the pooled prevalence of COVID-19 vaccine acceptance among study participants was 55.04% (95 % CI: 47.80–62.27 %), I^2^ = 99.55%. Being male [POR = 1.88 (95% CI: 1.45, 2.44)], having a positive attitude toward the COVID-19 vaccine [POR = 5.56 (95% CI: 3.63, 8.51)], having good knowledge in the COVID-19 vaccine [POR = 4.61 (95% CI: 1.24, 8.75)], having government trust [POR = 7.10 (95% CI: 2.37, 21.32)], and having undergone COVID-19 testing in the past [POR = 4.41 (95%CI: (2.51, 7.75)] were significant predictor variables.

**Conclusion:**

This analysis showed that respondents had a decreased pooled prevalence of COVID-19 vaccination acceptance. Sex, attitude, knowledge, government trust, and COVID-19 testing were statistically significantly correlated characteristics that affected the acceptability of the COVID-19 vaccine. All stakeholders should be actively involved in increasing the uptake of the COVID-19 vaccine and thereby reducing the consequences of COVID-19. The acceptance of the COVID-19 vaccination can be increased by using this conclusion as an indicator for governments, healthcare professionals, and health policymakers in their work on attitude, knowledge, government trust, and COVID-19 testing.

## Background

Coronavirus disease 2019 (COVID-19) is a dangerous communicable disease caused by infection with severe acute respiratory syndrome coronavirus 2 (SARS-CoV-2) (1). COVID-19 was first pronounced in Wuhan, China, in December 2019 (2). It has taken an enormous toll on the world's population through its mortality, morbidity, and impact on mental health and quality of life (3). Due to COVID-19's dissemination throughout the globe speedily, the World Health Organization (WHO) officially stated a pandemic and a public health emergency of international concern (PHEIC) (3). Even if the COVID-19 epicenters were in China, some European countries, and the United States (4), all other parts of the world, including Sub-Saharan Africa (SSA), are affected directly and indirectly by the pandemic (5). This is due to the large volumes of air transportation between these nations and Africa during the spread of COVID-19 (6). Besides, fragile health facility infrastructure, low clinician–population, inadequate laboratory competence, malnutrition, anemia, HIV/AIDs, and chronic respiratory diseases (7–9) led to worries that SSA could be hit hard. According to the WHO as of 22 June 2021, Africa has scored above 5.1 million cases with over 137,000 mortality (10). SSA is one of the least affected regions, with 28,848 diseased cases and 1,112 mortality recorded as of 27 April 2020 (11).

Consequently, enormous efforts by scholars, NGOs, and governments were absorbed in the development of successful and harmless vaccines for COVID-19 (12). Vaccination movements in high-income countries are fast-tracking in the world, but the population in low-income countries continues to be grievously exposed to COVID-19 (13). This gap is not only due to the inaccessibility of vaccines for COVID-19 but also due to the different perceptions of the community about the COVID-19 vaccine. Approximately 36% of South Africans (14), 6% of Ethiopians, and 41% of the population in the Democratic Republic of Congo are reluctant to be vaccinated against COVID-19 (15).

Many studies on COVID-19 vaccine acceptance and associated factors have been conducted in different parts of the world, including sub-Saharan African countries. These findings showed a variation in the magnitude of COVID-19 vaccine acceptance and its associated factors. Some shreds of evidence showed that COVID-19 vaccine acceptance was very low. There is a need to find reasons why COVID-19 vaccine acceptance differs in various sub-Saharan African countries, which enables stakeholders to formulate strategies for promoting COVID-19 vaccines in these regions. Therefore, this systematic review and meta-analysis were designed to estimate the pooled effect size and its predictor variables.

## Method

### Search strategies

The search results were reported based on the Preferred Reporting Items for Systematic Review and Meta-analysis statement (PRISMA) guideline (14).

PubMed, Embase, *MEDLINE*, Web of Science, Google Scholar, and Cochrane Library databases were used in comprehensive searching. The search words included “COVID-19” OR “SARS-CoV-2” OR “coronavirus” OR “novel coronavirus” OR “SARS-CoV-2” AND “vaccines” OR “vaccination” OR “COVID-19 Vaccines” OR “Vaccine acceptance” OR “level of COVID-19 acceptance”, “vaccination perception” OR “vaccine hesitancy” AND “associated factors” OR “Determinants” AND “Sub-Sahara African countries.” A manual search of the reference lists was also done to find possible important articles. All published articles were included.

### Screening of eligible studies

Two reviewers (EA and ME) conducted the screening using titles and abstracts of the articles independently. Any discrepancies in screening results were resolute by discussion between reviewers and, if needed, the overall team to reach a consensus. All screened studies were exported into EndNote version 8.1, and duplication records were removed. Then, these reviewers retrieved all the involved full-text articles. Then, the overlapping articles that both assessors chose were considered for the succeeding phase without any doubt, and disparities were solved with the help of the reviewers (JA and DZ).

### Inclusion and exclusion criteria

The inclusion criteria were as follows: (1) cross-sectional studies conducted among sub-Saharan African countries; (2) studies published in English language articles; (3) studies that investigated COVID-19 vaccine acceptance and/or associated factors; and (4) studies that used a standardized and validated tool. The exclusion criteria were as follows: (1) nonrelevant articles; (2) not fully reported; (3) low-quality studies; and (4) studies with an unsatisfactory value (0–4 points) were considered as low quality and excluded from the final systematic review and meta-analysis.

### Study quality assessment and data extraction

All studies were assessed for methodological quality by two independent reviewers using the reference to the following 10 items (clear statement of the aims of the research, methodology appropriateness, research design appropriateness, recruitment strategy, way of data collection, the relationship between researcher and participants, ethical issues, data analysis, statement of findings, and valuably of the research), from the Newcastle-Ottawa Scale (NOS) for analytical cross-sectional studies (15). The methodological quality of each included study was valued as very good quality (9–10 points), good quality (7–8 points), satisfactory (5–6 points), and unsatisfactory (0–4 points). The modified NOS for cross-sectional studies was used to include studies with ≥5 out of 10, which is considered a high-quality score (16). Based on the criteria, only high-quality studies were included.

Upon choosing the articles and appraising their quality, data in Table 1 were extracted. Author name(s), publication year, sample size, study population, study setting, outcomes, study design, and associated factors (pieces of information) were extracted from the included studies using Microsoft Excel 2016 (Table 1). Finally, the data were imported to STATA software version 14 for analysis.

**Table 1:** Characteristics of studies included for the final systematic review and meta-analysis of COVID-19 vaccine acceptance and/or associated factors (*n* = 29).

**SN**.	**References**	**SS**	**Country**	**V A (%)**	**Study setting**	**Target population**	**Study design**	**The final score of NOS**
1	Nzaji et al., 2020 (17)	613	Congo	27.7	Health facilities	HCWs	CS	7
2	Ditekemena et al., 2021 (18)	4,131	Congo	55.9	Community-based	General Population	CS	8
3	Ataboho et al.„ 2021 (19)	703	Congo	45.9	Community-based	General Population	CS	8
4	Tlale et al., 2022 (20)	5,300	Botswana	73.4	Community-based	General Population	CS	7
5	Ayele et al., 2021 (21)	422	Ethiopia	45.3	Health facilities	HCWs	CS	7
6	Berihun et al., 2021 (22)	416	Ethiopia	59.4	Health facilities	Chronic disease	CS	7
7	Mose et al., 2021 (23)	396	Ethiopia	70.7	Health facilities	Antenatal Care	CS	8
8	Taye et al., 2021 (24)	423	Ethiopia	69.3	School	Students	CS	8
9	Boche et al., 2022 (25)	319	Ethiopia	73	Health facilities	HCWs	CS	8
10	Agyekum et al., 2021 (26)	234	Ghana	39.3	Health facilities	HCWs	CS	8
11	Manan et al.„ 2022 (27)	348	Ghana	35	Community-based	General Population	CS	7
12	Lamptey et al., 2021 (28)	1,000	Ghana	54.1	Community-based	General Population	CS	8
13	Stephen et al., 2021 (29)	267	Ghana	41.95	Community-based	General Population	CS	7
14	Echoru et al., 2021 (30)	1,067	Uganda	53.6	Community-based	General Population	CS	8
15	Kanyike et al., 2022 (31)	600	Uganda	37.3	School	Students	CS	7
16	Yassin et al., 2022 (32)	793	Nigeria	97.3	Health facilities	HCWs	CS	8
17	Mustapha et al., 2021 (33)	440	Nigeria	40	School	Students	CS	7
18	Allagoa et al., 2021 (34)	1,000	Nigeria	24.6	Health facilities	Patients	CS	9
19	Yassin et al., 2022 (32)	400	Sudan	63.8	Health facilities	HCWs	CS	7
20	Raja et al., 2022 (35)	217	Sudan	55.8	School	Students	CS	8
21	Hoque et al., 2020 (36)	346	South Africa	63.3	Health facilities	Women	CS	8
22	Adeniyi et al., 2021 (37)	1,308	South Africa	90.1	Health facilities	HCWs	CS	9
23	Ngasa et al., 2021 (38)	371	Cameroon	45.38	Health facilities	HCWs	CS	7
24	Dula et al., 2021 (39)	1,878	Mozambique	71.4	Community-based	General Population	CS	8
25	McAbee et al., 2021 (40)	551	Zimbabwe	55.7	Community-based	General Population	CS	7
26	Mudenda et al., 2021 (41)	677	Zambia	33.4	Community-based	General Population	CS	8
27	Mudenda et al., 2022 (42)	326	Zambia	24.5	School	Students	CS	7
28	Kivuva et al., 2021 (43)	659	Kenya	51.3	Community-based	General Population	CS	7
29	Carpio et al., 2021 (44)	1,050	Kenya	96	Community-based	General Population	CS	8

SN, Serial number; PI, primary investigator; PY, publication year; SS, sample size; VA, Vaccine acceptance; HCWs, Healthcare workers; CS, Cross-sectional.

### Statistical analysis

COVID-19 vaccine acceptance and odds ratios of associated factors found in each article were aggregated after changing the original appraisals. To measure COVID-19 vaccine acceptance, point estimates of effect size, odds ratios (ORs), and 95% confidence intervals (95% CI) were calculated. Due to the variability of factors and heterogeneity of studies, the random effect model is more suitable for calculating the combined effects of COVID-19 vaccine acceptance (45).

Sub-group analysis also was done based on country, study setting, sample size, and year of publication to identify sources of heterogeneity. The degree of heterogeneity of these studies was measured qualitatively (forest plot) and quantitatively (I^2^ statistics, Cochrane's Q statistics). Then, values of I^2^ > 50% were considered an indication of high heterogeneity (46). Egger's weighted regression method was implemented for calculating the presence and effect of publication bias. All the analyses were conducted using statistical packages in STATA version 16.

### Protocol registration

The review protocol was registered with the PROSPERO database through a registration number (PROSPERO-CRD42022330781).

## Results

### Search results

**Figure 1:**
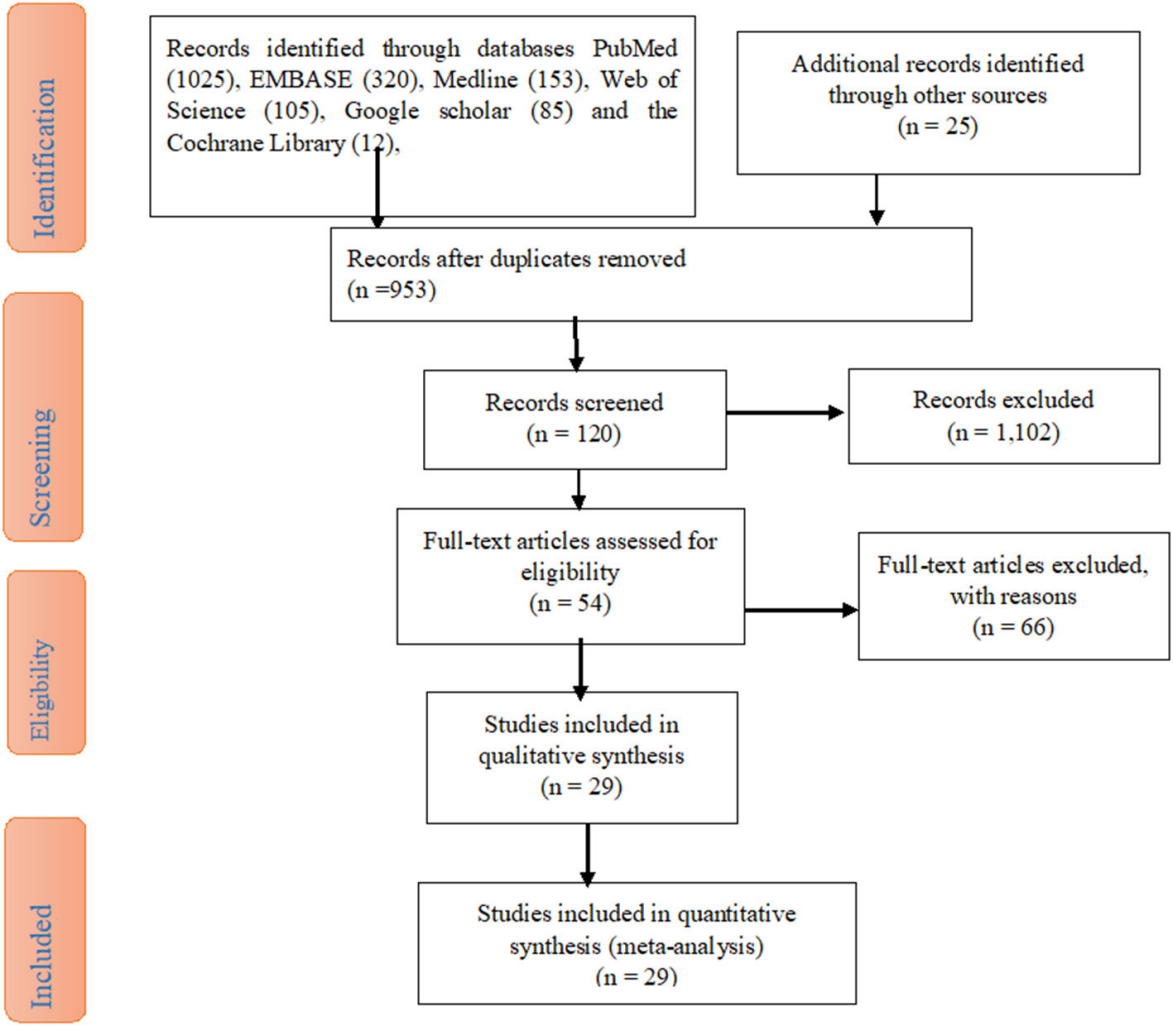
A PRISMA diagram of the study selection procedure on the acceptance of the COVID-19 vaccine in sub-Saharan Africa Countries.

The study search for the articles' results was reported according to the Preferred Reporting Items for Systematic reviews and Meta-Analysis (PRISMA) statement. After a compressive search through electronic databases such as PubMed (1,025), Embase (320), Medline (153), Web of Science (105), Google scholar (85), and the Cochrane Library (12), studies were recorded. Due to duplication, 953 records were removed. Through the assessment of the titles and abstracts, 1,102 articles were removed. Then, 120 studies were retrieved and 66 articles were excluded. Finally, 29 studies were included due to their eligibility criteria (Figure 1).

### Characteristics of included articles

Approximately 29 articles met the selection criteria and were included in this systematic review and meta-analysis. All articles had a cross-sectional design with data collected using face-to-face interviews, telephone, or online.

Five studies were from Ethiopia, four from Ghana, three from Congo, three from Nigeria, two from Uganda, Sudan, Zambia, Kenya, and South Africa, and one from Botswana, Cameroon, Mozambique, and Zimbabwe. These studies had 26,255 study participants ranging from 217 to 5,300, and the level of COVID-19 vaccine acceptance ranged from 24.5% (41) to 97.3% (47). The majority of the selected studies were community-based (18, 20, 28–30, 39, 40, 42–44), followed by the health institution (17, 21–23, 25, 26, 32, 34, 36–38, 47), whereas the remaining were school-based (24, 31, 33, 35, 41), considering the study setting. All of these studies were cross-sectional in study design (Table 1).

### Meta-analysis

#### Prevalence of COVID-19 vaccine acceptance

**Figure 2:**
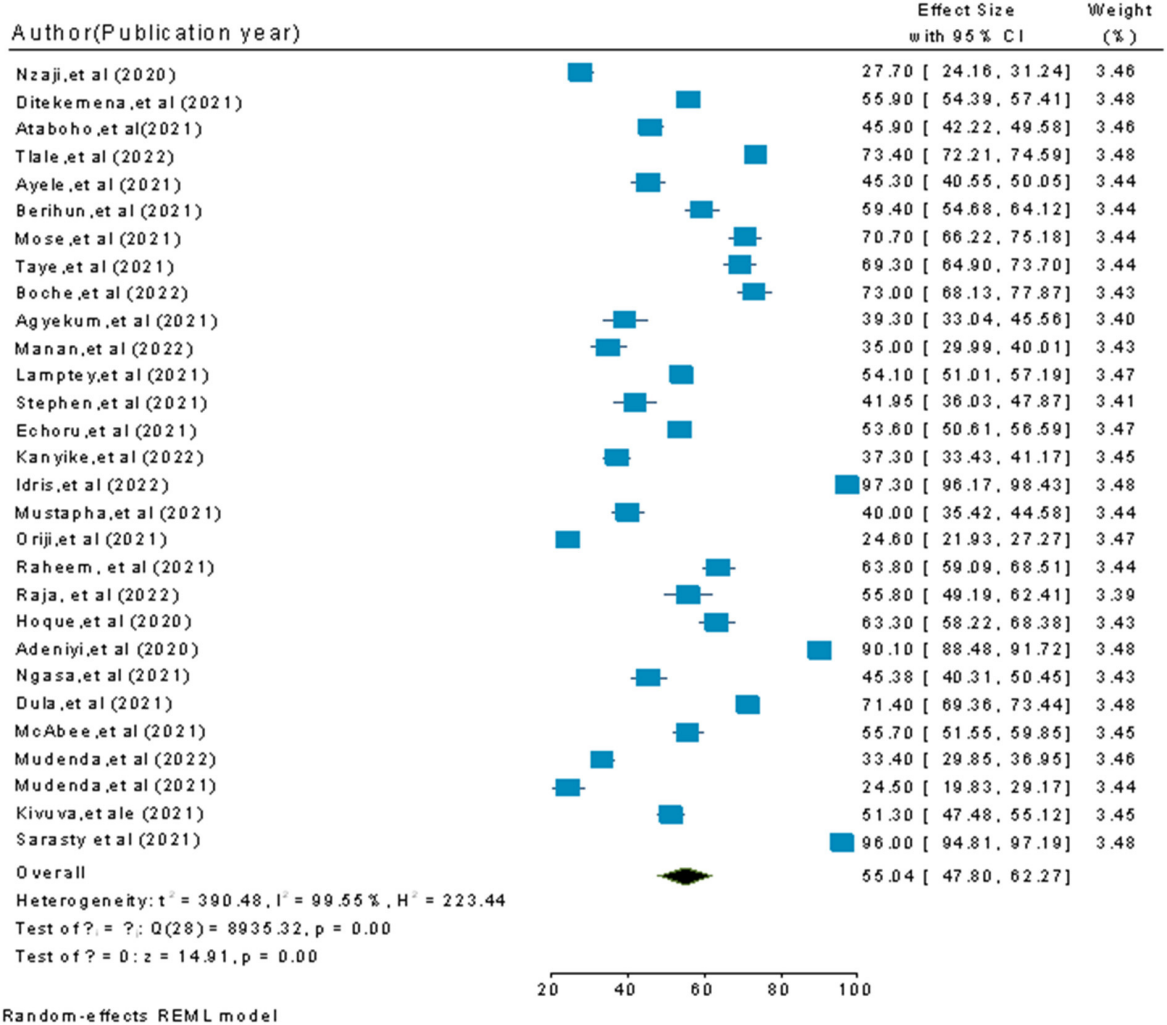
A pooled estimate of the COVID-19 vaccine acceptance rate among participants in sub-Saharan African Countries.

Since high heterogeneity was observed, a random-effects meta-analysis was conducted to assess the differences from an inverse-variance model. The pooled estimate of COVID-19 vaccine acceptance prevalence was 55.04% (95% CI: 47.80–62.27; *P-*value < 0.001) at a higher degree of heterogeneity (I^2^ = 99.55%) (Figure 2).

#### Publication bias

**Figure 3:**
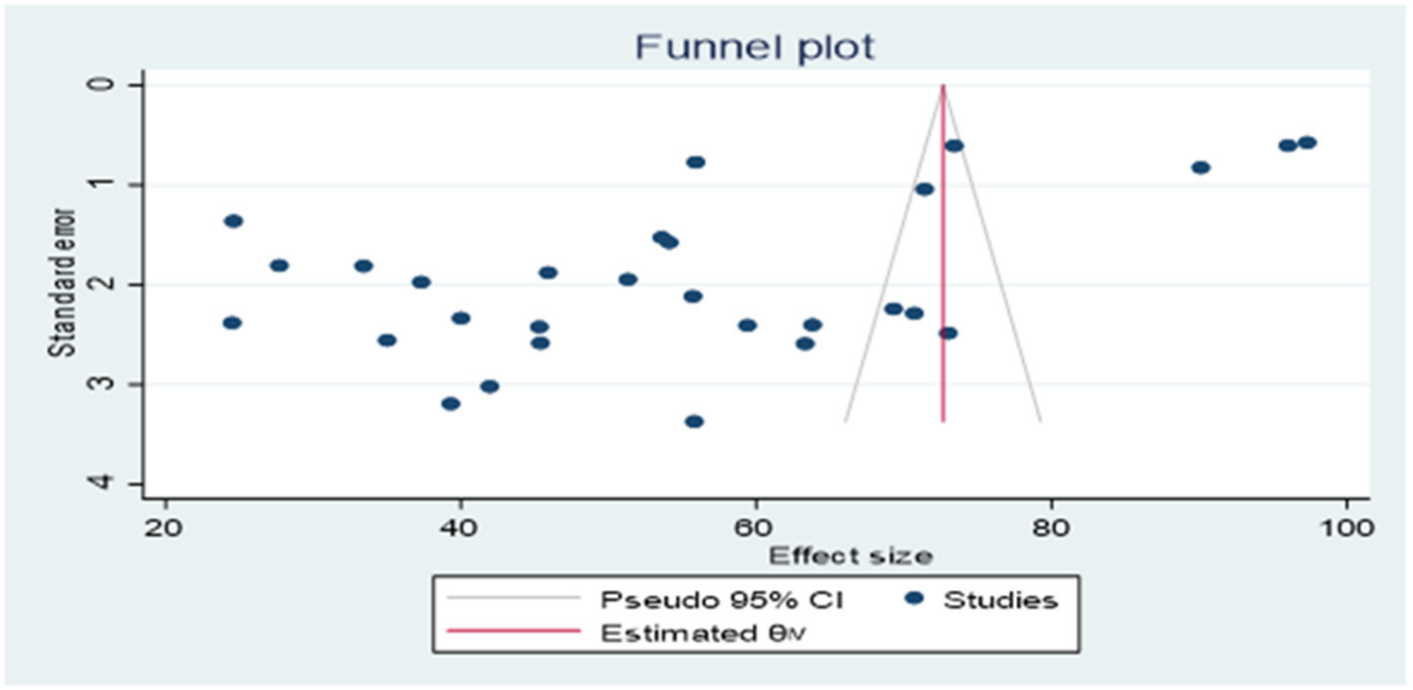
Assessment of publication bias.

Publication bias was observed based on the results of asymmetric examination using the funnel plot (Figure 3) followed by Egger's regression check (*P* = 0.0035).

**Figure 4:**
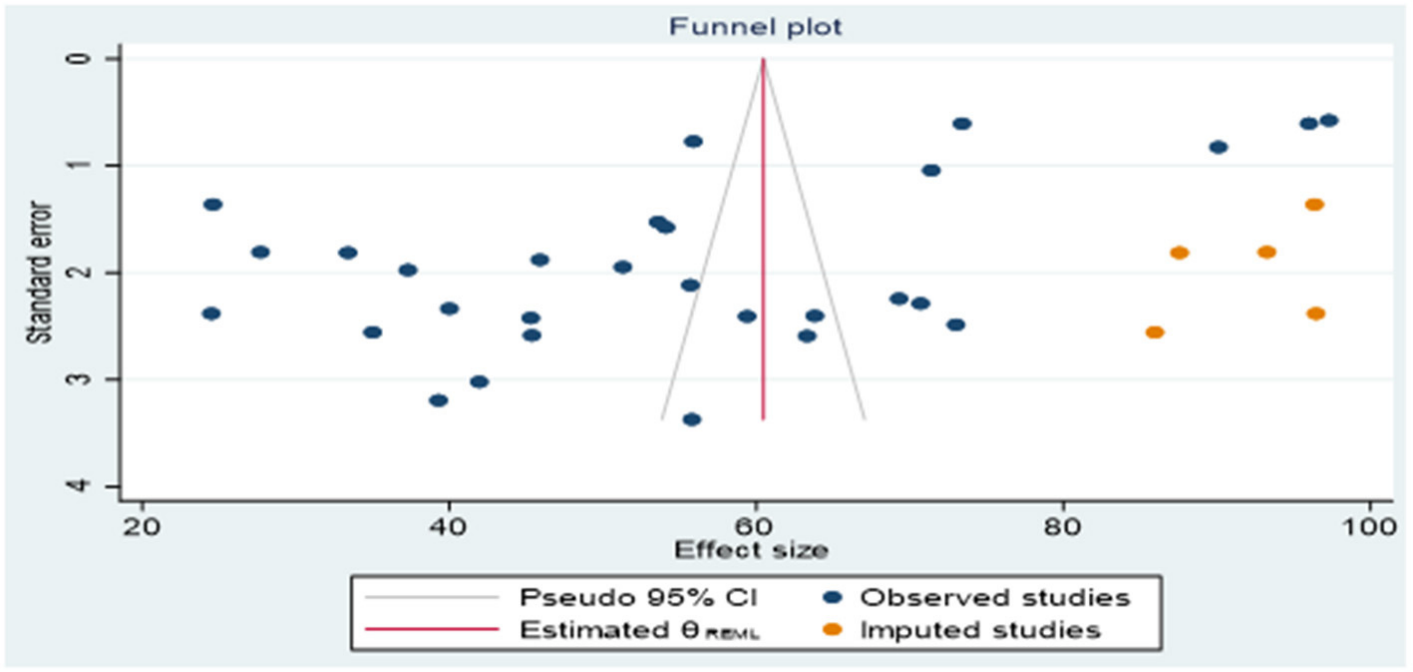
Funnel plot for publication bias after adjustment.

The result of the non-parametric trim-and-fill method indicated that, in addition to the 29 observed articles, there were an extra five important studies that were not observed because of publication bias (Figure 4). Therefore, our corrections change the number of significant results from imputing on the right from 55.04 to 60.453. However, there was no change from imputing on the right.

### Sub-group analysis of COVID-19 vaccination acceptance

Since the included studies varied in the study population, study period, study setting, effect of COVID-19, vaccine awareness, education status, and geographical areas, high heterogeneity was observed (I^2^ = 99.7%). Hence, subgroup analysis was done to find the source of heterogeneity. This was performed based on country, sample size, study setting, and publication year.

In the country-based sub-group analysis, the pooled estimate of COVID-19 vaccine acceptance among study participants was [43.22%, 95% CI (27.02, 59.42)] in DR Congo, [63.55%, 95% CI (53.53, 73.57)] in Ethiopia, [42.80%, 95% CI (34.44, 51.17)] in Ghana, [45.50%, 95% CI (29.52, 61.47)] in Uganda, and [53.99%, 95% CI (10.60, 97.39)] in Nigeria.

The prevalence of acceptance of the COVID-19 vaccine among study participants from health institutions was [58.36%, 95% CI (45.40, 71.33)], from community-based studies was [55.73%, 95% CI, (45.72, 65.74)], and from school-based studies was [45.34%, 95% CI (30.07, 60.62)].

The prevalence of acceptance toward the COVID-19 vaccine among studies published in 2020 was [60.39%, 95% CI (24.92, 95.86)], in 2021 was [53.12%, 95% CI (42.43, 60.81)], and in 2022 was [57.94%, 95% CI (39.80, 76.08)].

**Figure 5:**
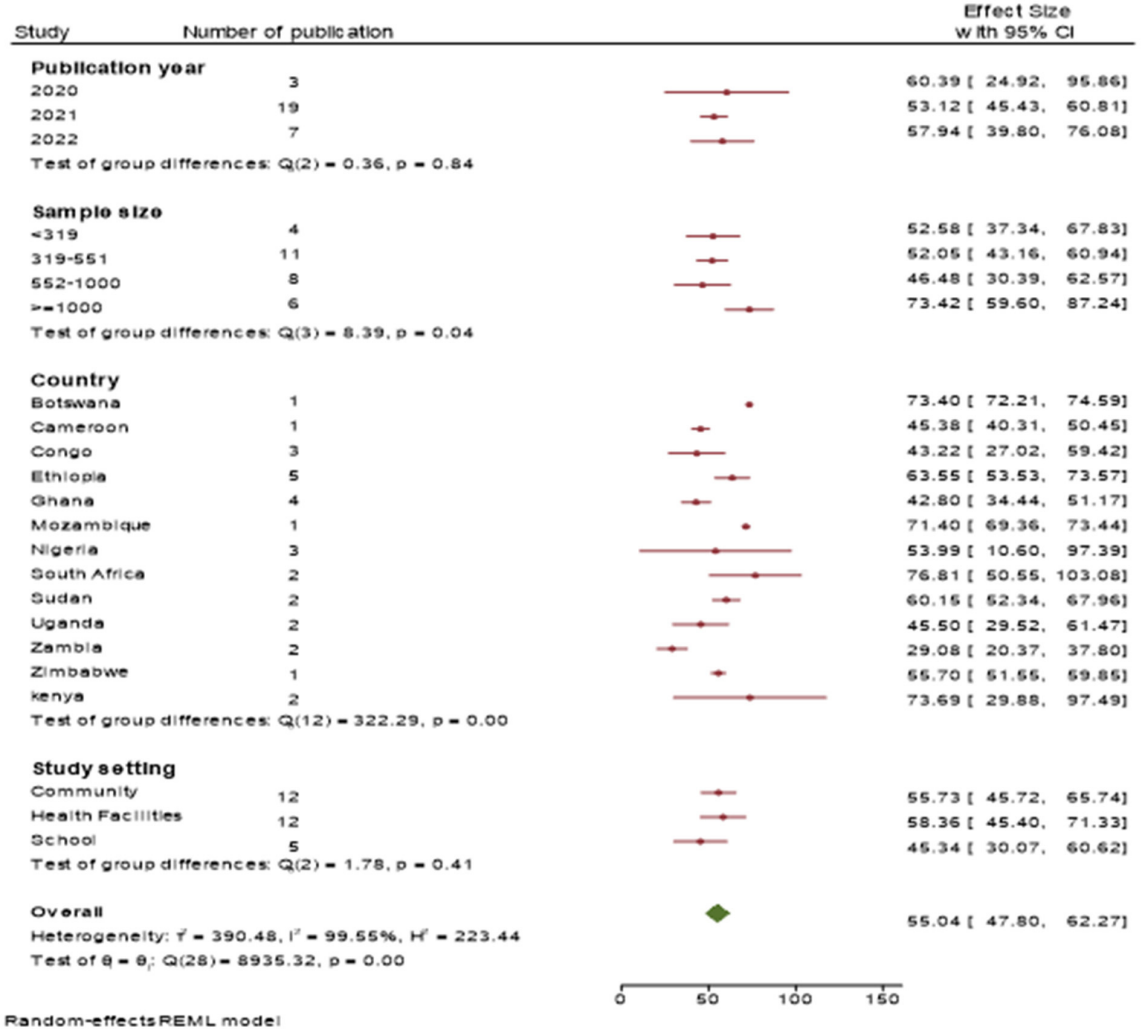
Forest plot showing the pooled prevalence of COVID-19 vaccine acceptance among the study population through subgroup analysis by sample size, country, study setting, and publication year.

The prevalence of acceptance of the COVID-19 vaccine among the respondents in studies with a sample size of < 319 was [52.58%, 95% CI (37.34, 67.83)], in studies with a sample size of 319–551 was [52.05%, 95% CI (42.16, 60.94)], in studies with a sample size of 552–1000 was [46.48%, 95% CI (30.39, 62.57)], and in studies with a sample size ≥1,000 was [73.42%, 95% CI (59.60, 87.24)] (Figure 5).

### Analysis of factors associated with COVID-19 vaccine acceptance

Sex, attitude, knowledge, government trust, and testing for COVID-19 were statistically significantly associated variables in COVID-19 vaccine acceptance.

**Figure 6:**
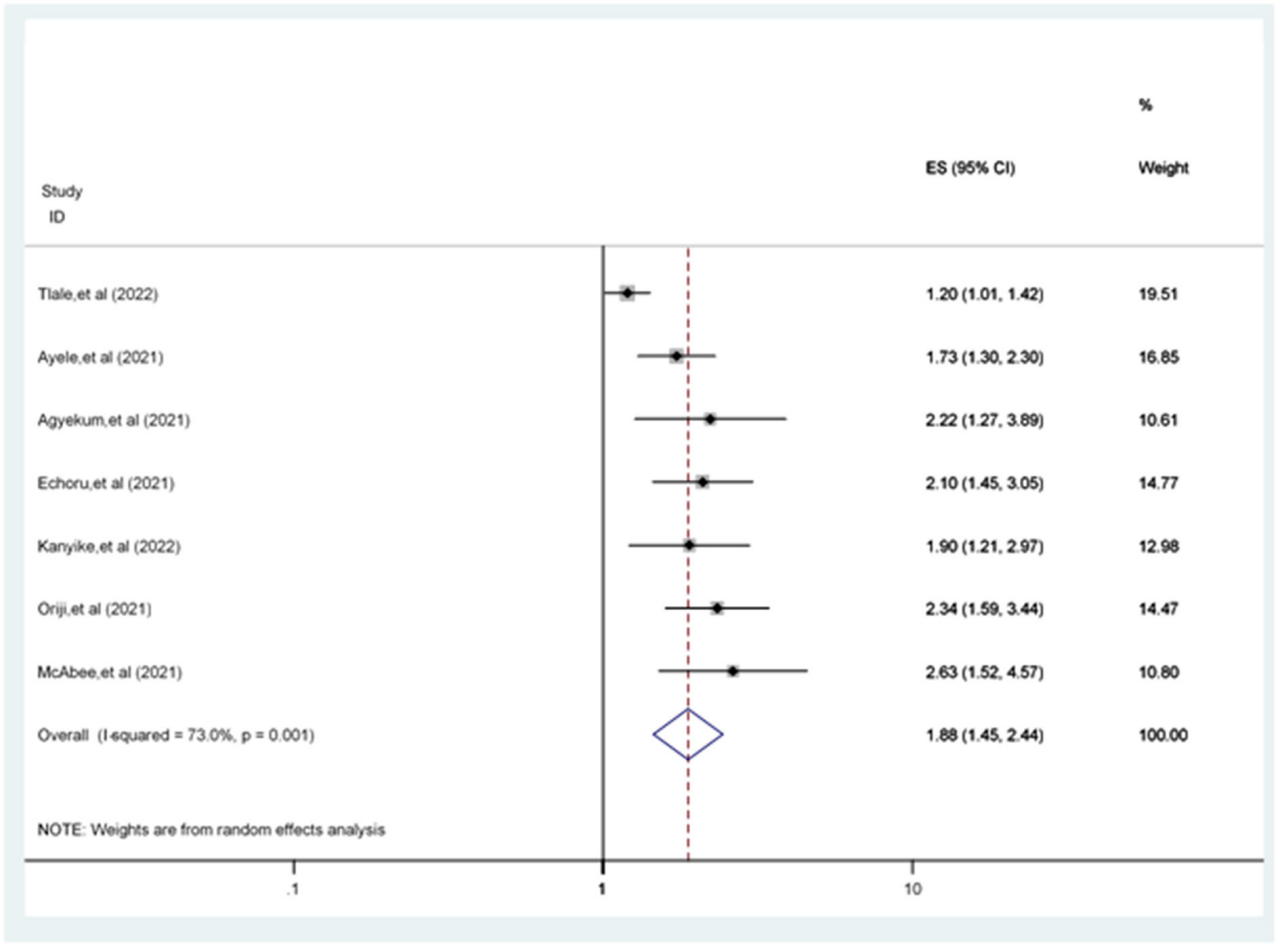
Forest plot revealing the relationship between sex and COVID-19 vaccine acceptance among the study population.

The odds of accepting the COVID-19 vaccine in men were 1.88 [POR = 1.88 (95% CI: 1.45, 2.44)] times more likely compared to women (Figure 6).

**Figure 7:**
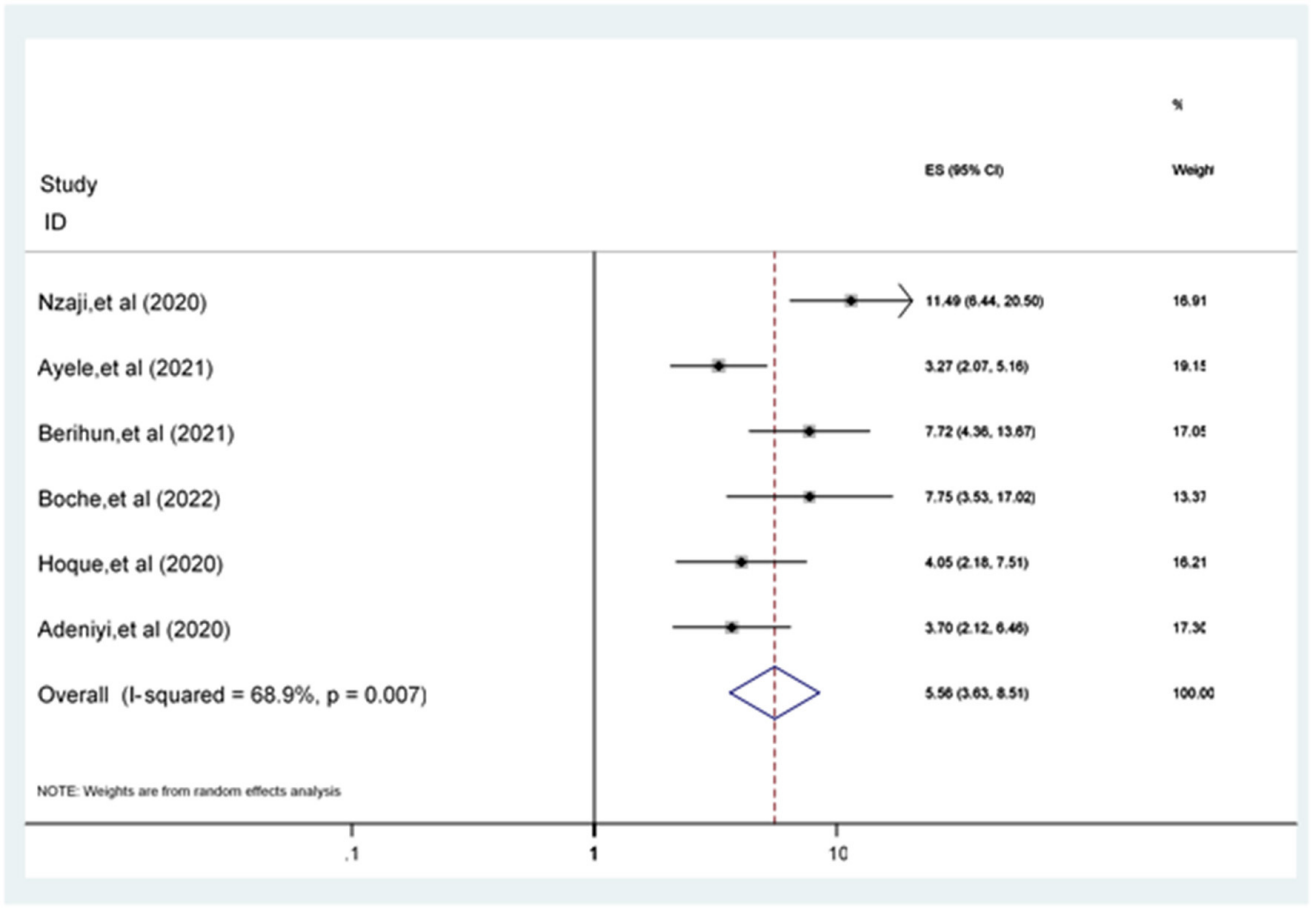
Forest plot revealing the relationship between attitude and COVID-19 vaccine acceptance among the study population.

Participants having a positive attitude toward the COVID-19 vaccine were 5.56 [POR = 5.56 (95% CI: 3.63, 8.51)] times more likely to accept the COVID-19 vaccine compared to participants who have a poor attitude (Figure 7).

**Figure 8:**
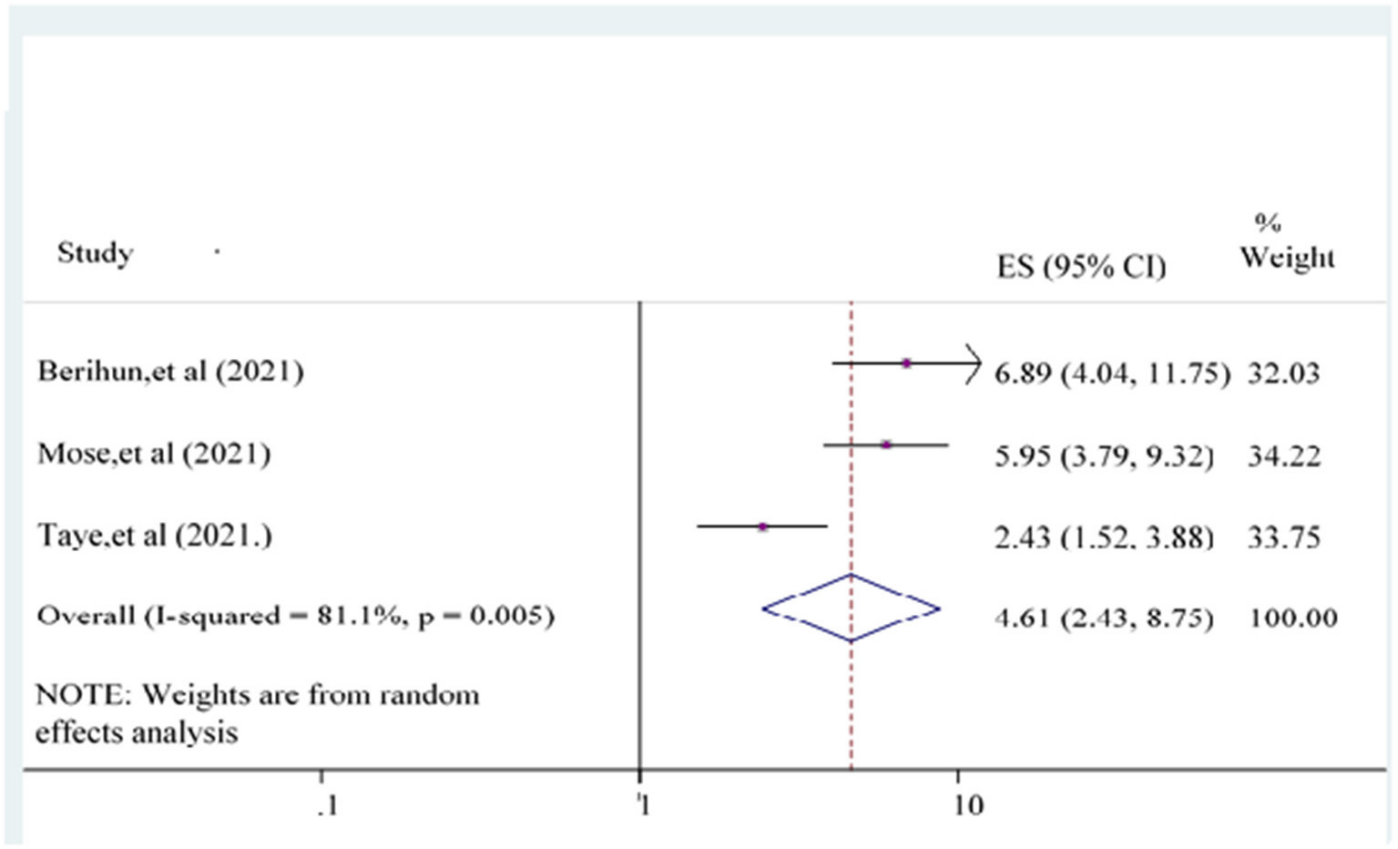
Forest plot showing the relationship between knowledge and COVID-19 vaccine acceptance among the study population.

The odds of having good knowledge of the COVID-19 vaccine {POR = 4.61 [95% CI: (2.43, 8.75)]} was 4.61 times more likely than poor knowledge counterpart participants (Figure 8).

**Figure 9:**
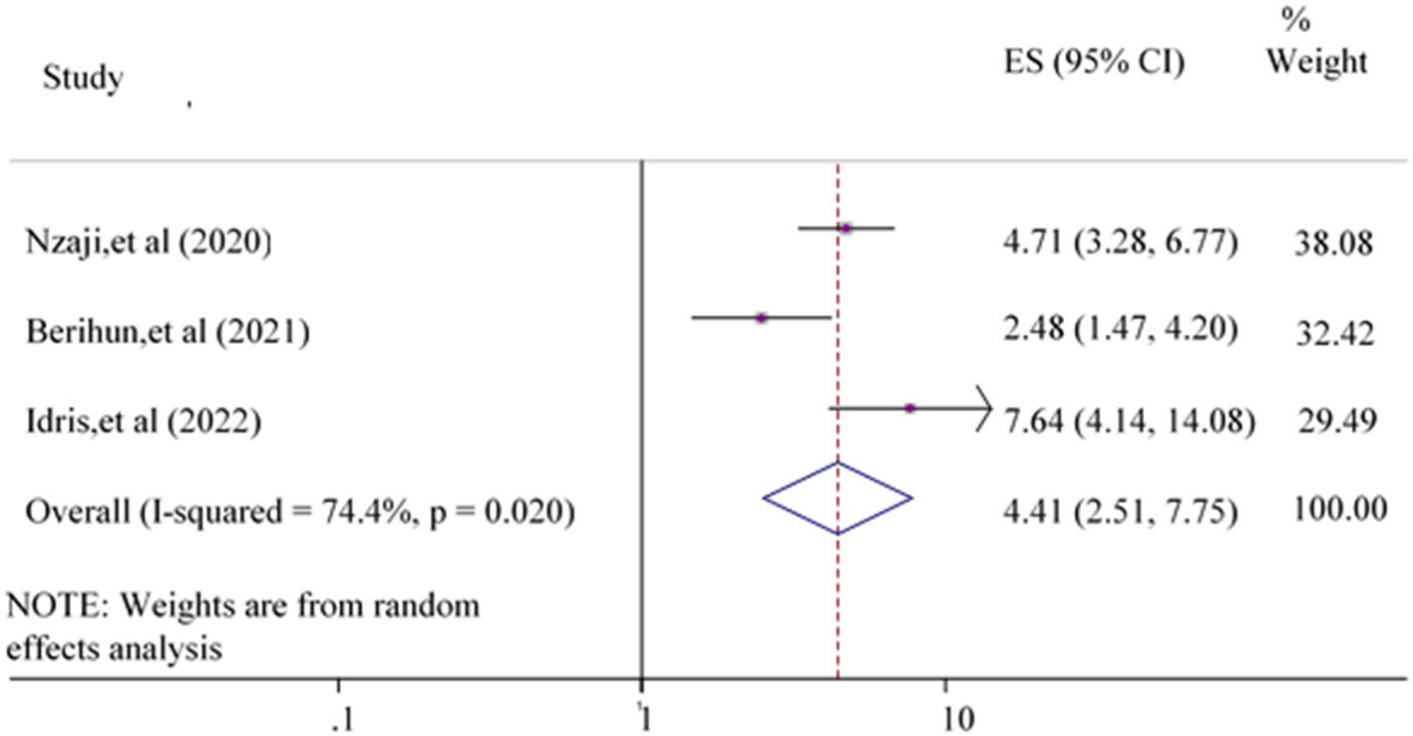
Forest plot showing the relationship between testing for COVID-19 and COVID-19 vaccine acceptance among the study population.

Whereas, participants previously tested for COVID-19 were 4.41 {POR = 4.41 [95% CI: (2.51, 7.75)]} times more likely to accept the COVID-19 vaccine compared to non-teased participants (Figure 9).

**Figure 10:**
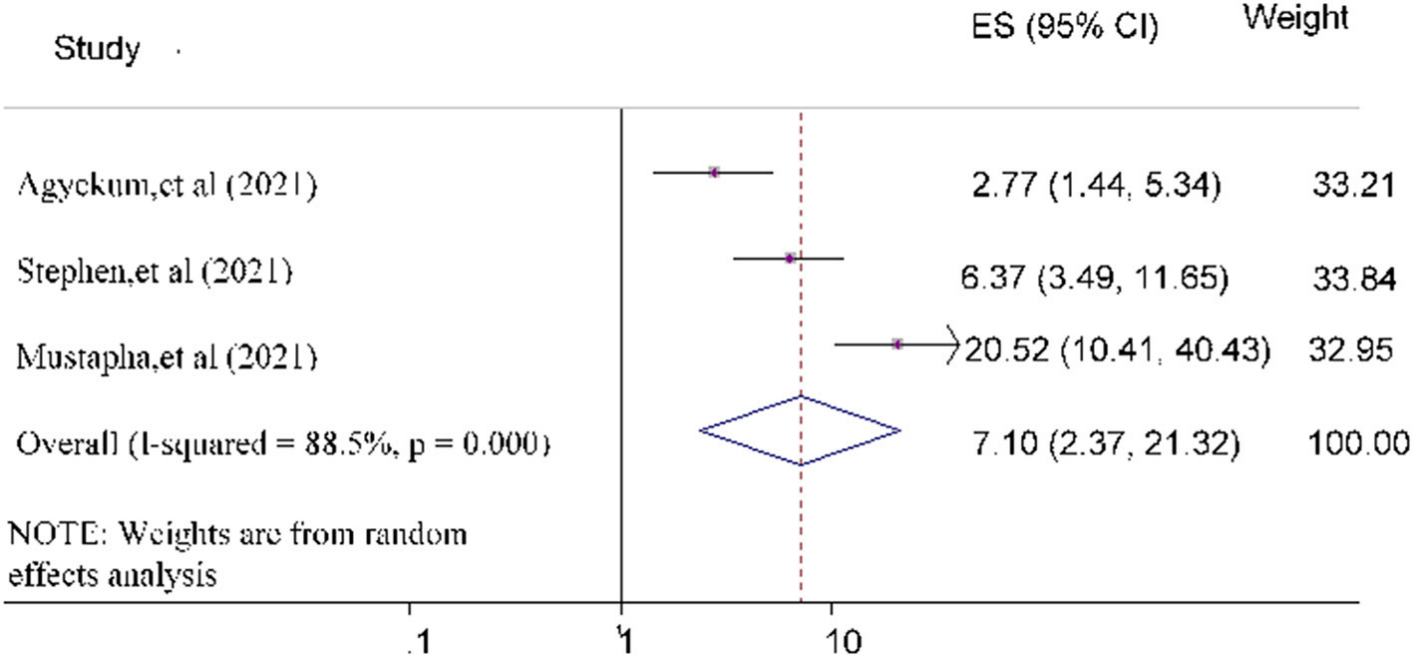
Forest plot depicting the relationship between government trust and COVID-19 vaccine acceptance among the study population.

Finally, the odds of government trust toward COVID-19 vaccine acceptance was 7.10 [POR = 7.10 (95% CI: 2.37, 21.32)] times more likely compared to study subjects who did not trust the government (Figure 10).

## Discussion

This systematic review and meta-analysis assessed COVID-19 vaccine acceptance and associated factors among sub-Saharan African countries. The pooled estimated prevalence of COVID-19 vaccine acceptance among sub-Saharan African countries was 55.04% (95% CI: 47.80–62.27%).

Our review findings showed that the acceptance of COVID-19 vaccines was lower than what was observed in previous national phone survey studies done among sub-Saharan African countries (87.6%) (48). This variation could be because the study subject chosen in the phone survey might not address the low-income people, people with no education, and others far from the media. This indicated that the phone surveys lacked representativeness from sub-Saharan African countries. Besides, the acceptance in this review was lower than the studies conducted on the global population (81.65%) (49), China (91.3%) (50), and Indonesia (93.3%) (51) and a review study (63.5%) (52). A possible explanation for this difference could be due to variations in the study setting, sociocultural characteristics, and study period.

However, this finding was better than the previous reviews on the pooled prevalence of COVID-19 vaccine acceptance in Africa (48.93%) (53). This disparity could be due to the dissimilarities in socio-culture, economic, level of awareness of the study participants, study setting, sample size, and study design toward the COVID-19 vaccine. This finding is comparable with low-income and lower-middle-income countries (58.5%) (3) and the global review (61.74%) (54) on COVID-19 vaccine acceptance.

This systematic review and meta-analysis indicated substantial variation in COVID-19 vaccine acceptance prevalence among study participants due to country, study setting, and year of publication difference. The subgroup analysis of this review showed that the highest COVID-19 vaccine acceptance prevalence was detected in South Africa (76.81%), while the lowest was observed in Zambia (29.08%). This lowest value of COVID-19 vaccine acceptance in Zambia is due to the uptake of the COVID-19 vaccine remaining unknown until 2021 (55). Another possible explanation for these differences could be the variation in media exposure, study setting, sample size, and sociodemographic profiles of the respondents.

The other characteristic used for subgroup analysis was the study setting. This subgroup analysis revealed that the highest COVID-19 vaccine acceptance was scored at health institutions [58.36%, 95% CI (45.40, 71.33)], followed by community-level study settings [55.73%, 95% CI (45.72, 65.74)], and the lowest COVID-19 vaccine acceptance prevalence was observed at school study setting [45.34%, 95% CI (30.07, 60.62)]. This disparity could be due to health institutions' study settings being more focused on healthcare workers who were more passionate about a COVID-19 vaccine than the general community and schools. This issue was supported by previous studies, which indicated that self-protection and readiness to safeguard families, friends, and sick people were significant predictors of overdue healthcare workers receiving vaccinations (56, 57). Meanwhile, healthcare workers have a more detailed understanding of COVID-19; consequently, they might be more likely to get the COVID-19 vaccine than the general population and school participants (55).

The pooled prevalence of COVID-19 vaccine acceptance among study participants in sub-Saharan African countries in 2020 was [60.39%, 95% CI (24.92, 95.86)], in 2021 was [53.12 %, 95% CI (42.43, 60.81)], and in 2022 was [57.94%, 95% CI (39.80, 76.08)]. This finding revealed that recent studies have higher COVID-19 vaccine acceptance. This difference might be due to the increased time people may be familiar with the COVID-19 vaccine through different communication media.

This review revealed a difference between men and women in COVID-19 vaccine acceptance rates. Based on seven studies (20, 21, 30, 31, 34, 40, 58), the odds of being male were higher in COVID-19 vaccine acceptance among study participants compared to the female participants. This finding is supported by earlier study evidence (49, 59). However, this finding contradicted a previous study (60). The possible explanation for this disparity might be that male participants are less familiar with protective and hygienic actions and are more exposed to different media, including Facebook. Male participants are more interactive with others, enhancing their information about COVID-19 vaccines, leading them to accept COVID-19 vaccines more than female participants (60).

Six studies (17, 21, 25, 26, 36, 37) examined attitude as a factor associated with accepting the COVID-19 vaccine. The pooled odds of a positive attitude toward the COVID-19 vaccine were a statistically significant predictor of COVID-19 vaccine acceptance. This result was in line with the studies conducted in Ethiopia (61), Korea (62), Indonesia (63), and China (50). Participants' positive attitude toward the safety and effectiveness of the COVID-19 vaccine looks to be the solution to the COVID-19 pandemic, especially the positive attitude concerning vaccination is essential (52).

Three studies (22–24) evaluated knowledge as a predictor of the acceptance of the COVID-19 vaccine. Participants with good knowledge about the COVID-19 vaccine were another significantly associated independent variable with COVID vaccine acceptance. This finding is supported by previous studies and surveys carried out in Ethiopia (61), England (64), and Southeast Asia (65). The possible explanation for this finding is that participants have good knowledge of using the COVID-19 vaccine and could accept the COVID-19 vaccine. The other reason might be that participants with good knowledge would be more informed and concerned about their health and wellbeing as a consequence of enhanced as well as becoming more concerned about life accomplishments that might affect them (55). Knowledge is the way for the directorial announcement in the strategies of escalating COVID-19 vaccine acceptance and for understanding means of organizing for future health crises (66).

Based on the evidence of three studies (26, 29, 33), government trust was a significant predictive factor in accepting the COVID-19 vaccine. Study participants with the highest confidence level in the national government had higher acceptance of the COVID-19 vaccine than their counterparts with the least trust. This finding is in line with other previous studies conducted worldwide (66–70). Political influences play a most important share in determining attitudes toward COVID-19 vaccination. Another possible explanation could be that trusting the government's competence in confirming that the COVID-19 vaccine may have fewer side effects and is effective, increasing their acceptance of the COVID-19 vaccination (71).

Finally, three articles (17, 22, 47) on the previous testing for COVID-19 were evaluated as a predictor of the acceptance of the COVID-19 vaccine. The odds of accepting the COVID-19 vaccine were higher among respondents who were tested for COVID-19 previously compared to participants who did not test for COVID-19. The possible reason for this difference might be that those respondents who participated in COVID-19 testing could create awareness of the COVID-19 vaccine and the consequences if they, their families, and the community at large are not vaccinated. Another possible explanation is that COVID-19 testing is not only for checking but also for educating the public about COVID-19 costs and the implications of the COVID-19 vaccine.

### Limitations of the review

First, the effect of the latent variable in the primary studies was not assessed, affecting the interpretation of the predictor variable in this review. Second, the other limitation, some important covariates, such as age, level of education, and comorbidity, were not reported sufficiently in many studies to include in this review. Third, the COVID-19 infection status during the review was not accessed.

## Conclusion

This review indicated that the pooled prevalence of COVID-19 vaccine acceptance was lower among respondents. Sex, attitude, knowledge, government trust, and testing for COVID-19 were statistically significantly associated factors toward COVID-19 vaccine acceptance; and they were also determinant factors in COVID-19 vaccine acceptance. All stakeholders should be vigorously involved to increase the acceptance level of the COVID-19 vaccine to prevent the impacts of COVID-19. This conclusion might be used as an indicator for governments, healthcare workers, and health policymakers in evaluating attitude, knowledge, government trust, and testing for COVID-19, which can improve COVID-19 vaccine acceptance.

## Data availability statement

The original contributions presented in the study are included in the article/supplementary material, further inquiries can be directed to the corresponding author.

## Author contributions

Data curation: JA, EA, and ME. Formal analysis, software, and writing: JA. Investigation, validation, methodology, and visualization: JA, DZ, EA, and ME. Writing–review and editing: JA, DZ, EA, ME, and VC. All authors contributed to the article and approved the submitted version.
